# Assessment and staff perceptions of mental health and substance use disorders integration in primary care

**DOI:** 10.1186/1940-0640-10-S1-A65

**Published:** 2015-02-20

**Authors:** Cheryl Teruya, Elise Tran, Darren Urada, Valerie P Antonini, Brandy Oeser, Katherine Lovinger

**Affiliations:** 1 Integrated Substance Abuse Programs, University of California, Los Angeles, CA, 90025, USA

## Background

Due to health-care reform, the integration of behavioral health (substance use disorder and mental health) services into primary care settings has taken on increased urgency. Project Care is an innovative county-funded integration initiative in Kern County, California, which uses a screening, brief intervention, and referral to treatment (SBIRT) model to build capacity for behavioral health care in two federally qualified health centers and one county medical center's outpatient clinic. Implementation strategies included funding to implement SBIRT reimbursement for the provision of two services (e.g., physical and mental health) in the same day, monthly provider meetings, trainings, technical assistance, and feedback in the form of evaluation reports to participating sites. As part of the program, assessments were conducted to measure integration progress.

## Methods

Repeated assessments of integration were conducted using the Dual Diagnosis Capability in Health Care Settings index (DDCHCS), which is a standardized organizational-level measure comprised of seven dimensions (e.g., program structure, clinical process—assessment, training), as well as surveys measuring staff perceptions of integration. Both types of assessments included baseline and yearly follow-ups at nine participating health center locations over the course of 3 years (2011–2014). A mixed model was used for analysis of the DDCHCS data to account for the repeated measures for each site. A generalized linear model with interaction terms was utilized in the analysis of staff survey data.

## Results

Significant improvements in participating organizations' DDCHCS overall mean scores were observed over time (p < .0001) after accounting for differences in organization, with the greatest change occurring between the baseline and first follow-up assessments. Similarly, average staff ratings of medical staff's effectiveness with behavioral health, communication between medical and behavioral health staff, and helpfulness of behavioral health services all showed significant increases (p < .05) from baseline to the first and second follow-up assessments.

## Conclusions

Most of the improvement in both the DDCHCS scores and staff survey ratings was observed between the baseline and first follow-up assessments. Overall, improvements shown at the first follow-up were maintained at the second follow-up. Results indicate that organizational capacity to deliver integrated services and staff satisfaction with integrated behavioral health care increased over time, and suggest that integration is feasible and acceptable.

